# Blood flow responses to different set pressures using elastic “multichambered” blood flow restriction bands

**DOI:** 10.3389/fspor.2026.1869097

**Published:** 2026-08-19

**Authors:** Pat R. Vehrs, Kalissa Cox, Sadie Heyborne, Emily Knote, Charlie Broberg, Jacob Clark, Kayla Swinson, Cameron Callahan, Hailey McDougal, Evan Miller

**Affiliations:** 1 Department of Exercise Sciences, Brigham Young University, Provo, UT, United States; 2 Department of Statistics, Brigham Young University, Provo, UT, United States

**Keywords:** arterial occlusion pressure, blood flow restriction training, multi-chambered cuffs, resistance training, vascular occlusion

## Abstract

**Introduction:**

The blood flow responses to blood flow restriction (BFR) with “multi-chambered” BFR bands are not well understood. This study investigated the blood flow responses to four set pressures in the arm and leg using B-Strong BFR bands.

**Methods:**

Ultrasound was used to measure unoccluded blood flow prior to measuring blood flow at each of four set pressures (200, 300, 400, 500 mmHg) in the arm and the leg in 60 participants (30 males; 30 females).

**Results:**

A set pressure of 500 mmHg was unable to occlude blood flow in both limbs in all participants. Blood flow at each set pressure was expressed relative to the paired measure of unoccluded blood flow. There were no significant sex differences in relative blood flow at each set pressure in either the arm (*p* = 0.7088) or the leg (*p* = 0.9920). There was a main effect for the blood flow responses between set pressures in the arm (*p* < 0.0001) and the leg (*p* < 0.0001). In both males and females, paired comparisons indicated that blood flow was significantly different between all set pressures (*p* < 0.05), except between 300 and 400 mmHg (*p* = 0.0752) in the arm and between set pressures of 200 and 300 mmHg in the leg (*p* = 0.2518).

**Discussion:**

The wide range of blood flow responses to each set pressure in both limbs presents challenges to determining an effective set pressure to use during BFR training and suggests the possibility of diverse outcomes with BFR training with B-Strong bands. Nevertheless, the wide range of blood flow responses does not preclude muscle adaptations as they are the result of multiple factors. The practical use of using elastic “multi-chambered” BFR bands requires further investigation to guide the determination of effective set pressures during exercise.

## 1 Introduction

Partial restriction of arterial blood flow into the arms or legs is used during strength training, aerobic training, neuromuscular stimulation, or passive rest (1, 2) to induce localized hypoxia and metabolic stress as a stimulus for muscle adaptations (3, 4). Combining controlled blood flow restriction (BFR) with low-load resistance training (20%–40% 1RM) results in similar muscular adaptations as training with higher loads and has applications in a variety of fitness, athletic, and clinical settings. Cuff pressure, or set pressure, and the corresponding reduction in blood flow, hypoxia, and metabolic stress are important metrics of training with BFR. At a given set pressure, the width of the cuff, cuff material, and bladder design have a profound effect on the blood flow responses to BFR. Rigid, nylon cuffs with single bladders are similar to cuffs used to measure blood pressure. These cuffs distribute pressure equally around the circumference of the limb. “Multi-chambered” cuffs, such as B-Strong bands (https://bstrongtraining, B-Strong Training Systems, Park City, Utah), are narrow bands (5–7 cm) made of soft, semi-elastic material. The inflatable bladder has multiple compartments, or chambers, that are interconnected with air channels that inflate the chambers simultaneously as a single unit (5). Inflation of these bands results in areas of non-uniform compression around the circumference of the limb (6, 7). The areas of non-uniform compression correspond to the gaps between the chambers produced by the interconnecting air channels.

When using single bladder nylon cuffs to apply BFR during exercise, the recommendation is to use an individualized set pressure equivalent to 40%–80% of the limb's previously measured arterial occlusion pressure (AOP) (1, 8–10). Because AOP varies with cuff width and design (11–13), the same cuff used to measure AOP should be used to apply BFR during exercise (1, 8–10). The use of soft, semi-elastic bands presents two specific challenges to the AOP-based approach of BFR training. First, the narrow width, soft, semi-elastic material, and “multi-chambered” bladder construction are designed to avoid complete arterial occlusion (5). Several studies have confirmed the inability of B-Strong bands to completely occlude arterial blood flow in the arm and leg at set pressures as high as 500 mmHg (7, 13–15). Thus, the AOP-based recommendations for BFR training do not apply to the use of B-Strong bands. Second, two previous studies have reported blood flow responses across a range of set pressures using B-Strong bands that present challenges to the selection of a set pressure to use during BFR training (14, 15). For example, Citherlet et al. (14) measured blood flow in the arms and legs of 11 subjects at a range of set pressures up to 400 mmHg. Only set pressures of 350 and 400 mmHg were able to reduce blood flow (mL/min) below resting (unoccluded) blood flow values. Citherlet et al. (14) did not report the actual resting blood flow, or relative blood flow (% of resting unoccluded blood flow) at each set pressure. The most recent study (15) reported relative blood flow responses (% of resting unoccluded blood flow) to BFR at set pressures up to 500 mmHg. In the arm and leg, relative blood flow was similar at set pressures of 200 mmHg and above in males and 250 mmHg and above in females. In the leg, only set pressures of 400 mmHg and above (males) and 450 mmHg and above (females) significantly reduced blood flow below resting unoccluded values.

As certified personal trainers, strength and conditioning practitioners, and clinicians (e.g., physical therapists) cannot use AOP-based recommendations when using the B-Strong BFR training system, further study of the blood flow response to set pressures recommended by the manufacturer is warranted. In addition, the previous study (15) reported blood flow responses to a range of set pressures using the B-Strong band relative to an initial resting unoccluded blood flow measured prior to measuring blood flow at the different set pressures. Measuring unoccluded blood flow prior to measuring restricted blood flow at each set pressure would better account for changes in resting unoccluded blood flow that may occur over the course of multiple blood flow measurements. Thus, the purpose of this study was to evaluate the blood flow responses to BFR using B-Strong “multi-chambered” bands at four set pressures relative to its paired resting unoccluded blood flow. This more appropriately reflects the reduction in blood flow associated with each set pressure. We hypothesized that an AOP is not achievable using B-Strong bands in the arm or the leg at a set pressure of 500 mmHg, and that compared to the paired resting unoccluded blood flow, there is no reduction in relative blood flow over a range of set pressures.

## 2 Methods

### 2.1 Experimental approach

We used Doppler ultrasound to evaluate the blood flow responses to BFR with B-Strong “multi-chambered” bands across four set pressures (200, 300, 400, 500 mmHg) in the dominant arm and the dominant leg. All data were collected in a temperature-controlled lab. Two trained investigators performed the ultrasound examinations and two other trained investigators acted as keyboard operators during the measurement of AOP and blood flow. During training, the inter- and intra-rater reliabilities of blood flow measurements among the trained investigators were >0.98. We first measured arterial occlusion pressure (AOP) of the brachial and superficial femoral arteries in each limb. We then made four paired measurements of resting unoccluded blood flow and blood flow at a randomly selected set pressure (200, 300, 400, 500 mmHg) in the arm and in the leg using a B-Strong multi-chambered blood flow restriction band. Subjects participated in this study on two non-consecutive days (48–72 h apart). All of the measurements on the arm were completed on one of the days, and all of the measurements on the leg were taken on the alternate day. The order of the limb being measured on the two days was randomized, as was the order of the blood flow measurements at each of the set pressures.

### 2.2 Participants

A convenience sample of 60 physically active young adults (30 males, 30 females) voluntarily participated in this study. Participants self-reported accumulating at least 30 min of moderate intensity physical activity 5 d/wk and performing muscle strengthening activities at least 2 d/wk but not currently training for any particular event. Superficial femoral artery AOP and blood flow at each of the set pressures were measured in all 60 participants. Eight participants opted out of one of the days of participation due to availability or other personal reasons, so brachial artery AOP and blood flow at each set pressure were measured in 52 of the participants (26 males, 26 females). Exclusion criteria were females who were pregnant, less than six months postpartum or on oral birth control, self-reporting risk factors for cardiovascular diseases or thromboembolisms, a personal history or diagnosis of, or being treated for deep vein thrombosis or pulmonary embolism, hypertension, cardiovascular disease, diabetes, or renal disease. Females completed their participation in this study at any point during their menstrual cycle (16). Participants were instructed to refrain from eating within 2 h and consuming caffeine within 8 h prior to their participation. Participants were also asked to avoid planned exercise and vigorous physical activity during the 24 h prior to their participation. After the methods, expectations, risks and benefits of the study were explained, each participant provided written informed consent. This study was reviewed and approved by the Institutional Review Board prior to data collection.

#### 2.2.1 Participant characteristics

Body mass (kg) was measured using a digital scale (Ohaus Model CD-33, Ohaus Corporation, Pine Brook, NJ, USA) and height (cm) was measured using a calibrated stadiometer (SECA Model 264; SECA, Chino, CA, USA). Body mass and height were used to calculate body mass index (BMI; kg/m^2^). Circumferences of the self-reported dominant arm and leg measured in triplicate using a spring-loaded Gullick measuring tape were used to calculate the volume (m^3^) of the upper arm and thigh as previously described (17, 18). Participants then rested for 5 min in a semi-reclined seated position on a patient table with the legs extended (0° knee extension) and supported. Resting heart rate (HR) and blood pressure were measured using an automated device (OMRON BP7200, OMRON Healthcare, Inc.). Mean arterial blood pressure (MAP) was calculated using the measured brachial systolic (SBP) and diastolic (DBP) blood pressures.

### 2.3 Arterial occlusion pressure

An inflatable 10 cm wide, single bladder, straight nylon blood flow restriction cuff (https://hpluscuff.com/; Innovative Therapy Solutions, Santa Barbara, CA) was used to measure AOP of the brachial artery and the superficial artery. The AOP was measured on the same day as blood flow measurements on the same limb. The length of the cuff used to measure AOP for each participant was based on the limb circumference. The cuff was placed on the upper arm or upper thigh so that the center of the cuff bladder was positioned over the brachial artery or superficial femoral artery (19, 20). We used Doppler ultrasound with a 9 MHz linear array probe and a LOGIQ Fortis ultrasound machine (GE Healthcare, Chicago, IL, USA) to measure AOP. The AOP of the brachial artery was measured using ultrasound to detect blood flow in the radial artery just below the elbow with the arm in the supinated position. When measuring the AOP of the superficial femoral artery, we detected blood flow distal to the cuff on the medial side of the thigh. The location of the vessel was marked on the skin for reference during the subsequent measurement of blood flow. The blood flow restriction cuff was inflated using a hand-held pump and pressure gauge. The cuff was initially inflated to 50 mmHg, followed by a gradual increase in set pressure (≈10 mmHg/10 s) until color flow and pulse waves were no longer detected using ultrasound. To ensure an accurate measurement of AOP, the cuff was slowly deflated until the return of blood flow was confirmed by the presence of color flow and pulse waves. The cuff was then slowly reinflated to confirm the AOP. The AOP was defined as the lowest pressure at which color blood flow and pulse waves were no longer visible on the ultrasound display. After the AOP was recorded, the cuff was completely deflated and removed. Participants had a 5 min quiet rest period following the measurement of AOP.

### 2.4 Blood flow

The size of a B-Strong band to use is based on the circumference of the limb. The manufacturer recommended B-Strong band was placed on the arm or leg. With the band in place but not pressurized, ultrasound was again used to initially locate the radial artery or the superficial femoral artery at the previously marked location using a transverse view and then a longitudinal view. Blood flow was verified using color flow and pulse wave modes. The keyboard operator adjusted the gain, depth, fine steer, SV length, scale, and sweep speed to ensure the best image quality. The angle of insonation of the ultrasound probe was internally maintained at 60°. Once a clear image of the vessel was obtained, a cine-loop (video clip) that included 10–12 pulse waves was manually captured and saved for later analysis. Immediately after the recording of the cine-loop, the cuff was inflated to a randomly selected set pressure (i.e., 200, 300, 400, or 500 mmHg) using a hand pump and pressure gauge (range 0–500 mmHg) included with the B-Strong BFR system. To allow adequate time for hemodynamic responses to stabilize, the band remained inflated for 1 min before recording a cine-loop that included 10–12 pulse waves (14). The band was then deflated. After a 5 min quiet rest period (11, 12, 21) with the band in place but not inflated, the process was repeated with each of the remaining set pressures in a randomized order. A cine-loop of unoccluded blood flow was recorded prior to recording a cine-loop of restricted blood flow at each set pressure. Thus, the blood flow measurement from the cine-loop obtained at each set pressure was paired with its corresponding measurement of unoccluded blood flow. Cine-loops were saved using a standardized labeling system to ensure proper identification of each video during later analysis. During the analysis of each cine-loop, pulse waves were used as a reference point for the measurement of vessel diameter. A previous study (15) reported that measurements of blood flow when the vessel diameter was measured during systole, diastole, or averaged between measurements taken at systole and diastole, were similar. In this study, the vessel diameter was measured perpendicular to the direction of the blood flow during systole. Time averaged velocity (cm/s) of blood flow was recorded over 10 pulse waves. Blood flow (mL/min) was automatically calculated by the ultrasound machine. Because we used a standardized labeling system to correctly identify cine-loops, the trained investigator analyzing the cine-loops was not completely blind to the limb or pressure conditions.

### 2.5 Statistical analysis

We used an independent *t*-test to evaluate sex differences in participant characteristics of age, height (cm), body mass (kg), BMI (kg/m^2^), resting systolic (SBP), diastolic (DBP), and mean (MAP) blood pressures and heart rate (bpm). A familywise *p*-value of 0.05 was maintained using a Bonferroni corrected *p*-value = 0.0062 to account for multiple comparisons. Likewise, an independent *t*-test was used to evaluate sex differences in limb characteristics of limb circumference (cm), limb volume (m^3^), skinfold thickness (mm) and AOP (mmHg) of the arm and the leg. A familywise *p*-value of 0.05 was maintained using a Bonferroni corrected *p*-value = 0.0125 to account for multiple comparisons.

The primary data of interest in this study were the blood flow measurements taken in the radial and superficial femoral arteries at the four set pressures. Blood flow was expressed in relative terms (% of the paired unoccluded blood flow). Separate analyses were performed for the arm and leg. Blood flow data were analyzed using the “R” statistical analysis system (22) and the “lmer” (23) package. Denominator degrees of freedom were determined using the robust methods of Kenward-Rogers (24). To understand the relationship between relative blood flow and BFR set pressure, linear mixed-effects models with random intercepts were fit to the data. Set pressure was modeled as a categorical variable, sex and pressure were considered fixed effects and subjects were considered a random effect. A mixed model was used to appropriately account for multiple sources of variability. Standard diagnostic plots did not show any concerns about violations of the assumptions. When the mixed model showed significant main effects, Tukey adjusted paired comparisons of blood flow were made between all levels of covariates. A *p*-value of 0.05 was used to determine significance of paired comparisons.

## 3 Results

### 3.1 Participant characteristics

On average (mean ± SE), male participants were taller (180.2 ± 1.3; 167.7 ± 1.2 cm; *adj p* < 0.008), heavier (78.9 ± 2.4; 67.3 ± 1.7 kg; *adj p* = 0.008) and had higher systolic blood pressures (120.8 ± 2.0; 112.5 ± 1.9 mmHg; *adj p* = 0.040) than their female counterparts, respectively. There were no sex differences in age (25.8 ± 1.8; 22.2 ± 0.5 yr; *adj p* = 0.480), BMI (23.8 ± 0.7; 24.2 ± 0.7 kg/m^2^; *adj p* = 0.990), diastolic blood pressure (76.0 ± 1.8; 76.9 ± 1.4 mmHg; *adj p* = 0.990), mean arterial pressure (90.9 ± 1.6; 88.8 ± 1.1 mmHg; *adj p* = 0.990), or resting HR (68.1 ± 1.5; 71.7 ± 1.6 bpm; *adj p* = 0.784). Arm circumference (31.1 ± 0.8; 28.8 ± 0.7 cm; *adj p* = 0.148) and volume (0.0390 ± 0.002; 0.0327 ± 0.002 m^3^; *adj p* = 0.160) were similar in males and females, respectively. However, females (16.2 ± 1.7 mm) had a greater (*p* < 0.004) skinfold thickness than males (8.0 ± 1.4 mm). Likewise, leg circumference (57.6 ± 1.1; 59.6 ± 1.1 cm; *adj p* = 0.804) and volume (0.2171 ± 0.0110; 0.2168 ± 0.009 m^3^; *adj p* = 0.990) were not significantly different between males and females, respectively, but females (29.4 ± 2.3 mm) had a greater (*p* < 0.004) skinfold thickness than males (17.1 ± 1.8 mm). Sex differences in brachial artery (127.5 ± 2.9; 119.5 ± 2.5 mmHg; *adj p* = 0.188) and superficial femoral artery (200.6 ± 4.6; 209.7 ± 5.6 mmHg; *adj p* = 0.864) AOP were not significantly different between males and females, respectively.

### 3.2 Blood flow responses

**Figure 1:**
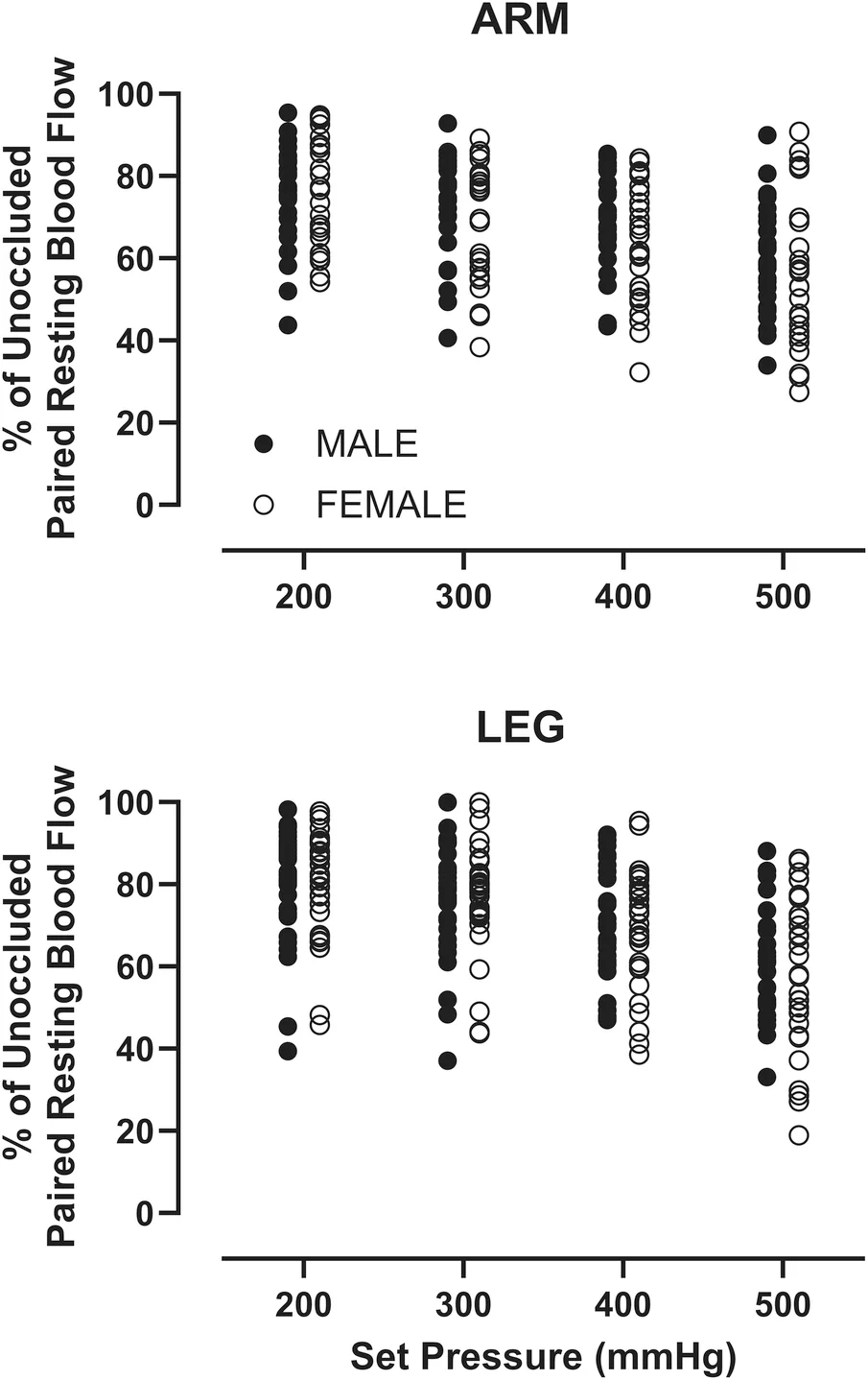
Individual relative blood flow (% of paired unoccluded blood flow) responses to increasing set pressures in the arm (upper graph) and leg (lower graph).

**Figure 2:**
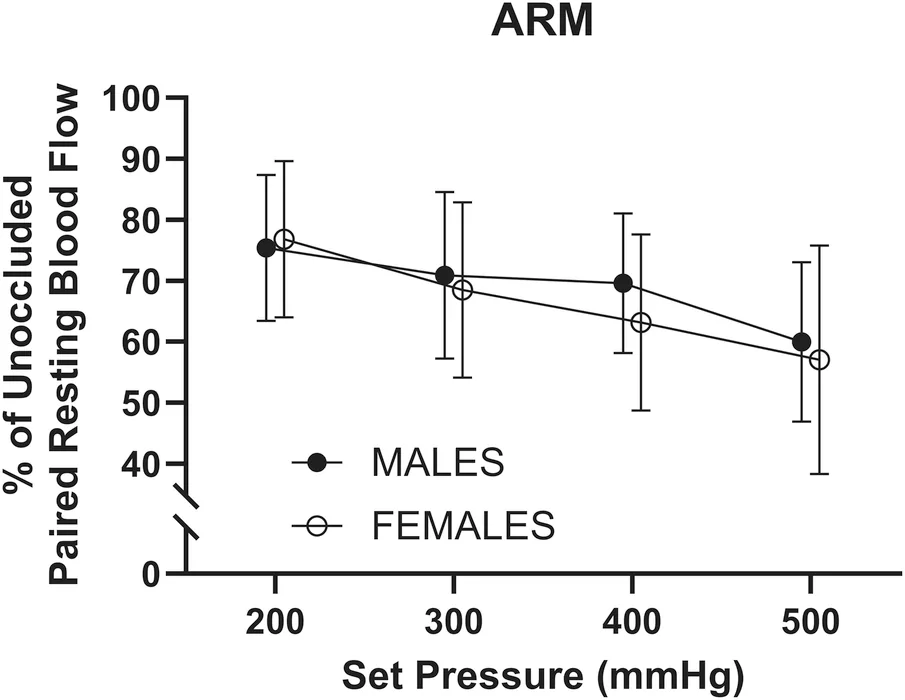
Relative blood flow (% of paired unoccluded blood flow) (mean ± standard deviations) in the radial artery. There are no significant differences in relative blood flow between males and females at any set pressure (*p* = 0.7088). Relative blood flow is similar at set pressures of 300 and 400 mmHg (*p* = 0.0752).

There was a wide range of individual blood flow responses to each of the set pressures in the arm and the leg (Figure 1). Relative blood flow in the arm (% of paired unoccluded blood flow) for male and females is illustrated in Figure 2. The mixed model analysis indicated that there was no overall effect of sex (df = 86.99, 95% C.I. = −6.1 to 9.0; *t* = 0.375, *adj p* = 0.7088), but there was an effect of set pressure (*t* > 3.87; df = 156; *adj p* < 0.0001). The sex by set pressure interaction was not significant (chi-sq = 6.6, df = 3; *adj p* = 0.0853). In both males and females, paired comparisons indicated that blood flow was significantly different (*adj p*-values < 0.05) between all set pressures, except between 300 and 400 mmHg (females df = 162, *t* = −2.432, *adj p* = 0.0752; males df = 162, *t* = −0.587, *adj p* = 0.9360).

**Figure 3:**
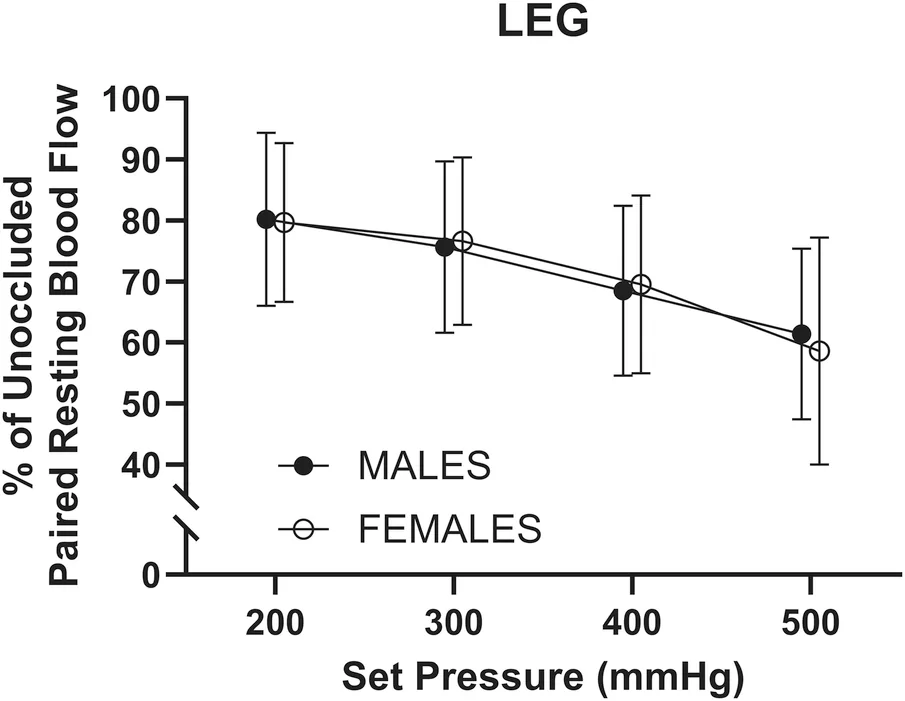
Relative blood flow (% of paired unoccluded blood flow; mean ± standard deviations) in the superficial femoral artery. There are no significant differences in relative blood flow between males and females at any set pressure (*p* = 0.992). Relative blood flow is similar at set pressures of 200 and 300 mmHg (*p* = 0.2518).

Relative blood flow (% of paired unoccluded blood flow) for male and females in the leg is illustrated in Figure 3. The mixed model analysis indicated that there was not an effect for sex (95% C.I. = −7.7 to 7.6; df = 116.9, *t* = −0.010, *p* = 0.992), but there was an effect of set pressure (0.812 ≤ *t* ≤ 8.80; df = 180; *p* < 0.0001). The sex by set pressure interaction was not significant (chi-sq = 1.65, df = 3; *p* = 0.4487). In both males and females, all paired comparisons of blood flow were significant (tukey adj *p-values* < 0.05), except for between set pressures of 200 and 300 mmHg (females df = 186, *t* = −0.798, *p* = 0.8552; males df = 86, *t* = −1.823, *p* = 0.2656).

## 4 Discussion

A recent review of the use of elastic B-Strong bands emphasized the importance and need for continued research rigor and the refinement of BFR methodologies and standardization of practice in the landscape of BFR training (5). The results of this study expand on previous reports (14, 15) of blood flow responses to BFR using B-Strong bands in the arm and leg. Contrary to the two previous studies, we report relative blood flow responses (% of paired unoccluded blood flow). This appropriately accounts for individual differences that are not accounted for when expressing blood flow in absolute terms and changes in resting unoccluded blood flow that may occur over time and across multiple measurements of blood flow. We also confirm the inability of B-Strong bands to achieve complete arterial occlusion in the brachial and superficial femoral artery.

### 4.1 Arterial occlusion pressure

A set pressure of 500 mmHg using the B-Strong band did not achieve complete occlusion of blood flow in the brachial or superficial femoral artery (Figure 1). Because an AOP was measured in the arm and leg using a single bladder nylon cuff, the inability of the B-Strong bands to achieve complete occlusion is due to the thick, soft, elastic material and barrel- or accordion-like construction that was designed to avoid complete arterial occlusion and limit peak pressures (5). At a set pressure of 500 mmHg, relative blood flow (% of paired unoccluded blood flow) was on average 58% (range from 27% to 90%) in the arm and 60% (range 18%–88%) in the leg (Figure 1). This concurs with a recent study (15) that reported that at a set pressure of 500 mmHg, blood flow in the arm was on average 51% (range 11%–75%; men) and 43% (range 16%–73%; women) of unoccluded blood flow. At a set pressure of 500 mmHg, blood flow in the superficial femoral artery was on average 60% (range 43%–83%; men) and 51% (range 19%–86%; women) of unoccluded blood flow (15). Although B-Strong bands were designed to avoid peak pressures (5), some subjects in this study experienced high levels of BFR. For example, the lowest relative blood flow (% of paired unoccluded blood flow) in the arm and leg was 27% (a 73% reduction in blood flow) and 18% (an 82% reduction in blood flow), respectively. By comparison, the data presented by Crossley et al. (25) indicates that at the set pressures recommended for use during AOP-based BFR training (40%–80% of AOP), superficial femoral artery blood flow was approximately 50%–60% of unoccluded resting blood flow.

Of concern is the use of practices, either by practitioners, BFR enthusiasts, or research groups, when using soft elastic multi-chambered bands that are appropriate only when using rigid nylon, single-bladder cuffs. For example, inflating B-Strong bands to a set pressure estimated from methods ascribed for a rigid nylon, single bladder cuff (26). Likewise, inflating B-Strong bands to the same absolute set pressure for all participants without explanation or regard for the effects of cuff and bladder design on blood flow responses to BFR (27, 28) should be avoided.

### 4.2 Relative arterial blood flow in the arm and leg

In both the arm and leg, there was not a difference in relative blood flow (% paired unoccluded blood flow) between males and females at any of the set pressures. This is contrary to the sex differences in relative blood flow in the arm and leg recently reported (15). The discrepancy between the data reported in this study and the previous study (15) can be explained, in part, by differences in sample size (20 vs. 60 participants), and that in the previous study, blood flow at each of the set pressures was expressed relative to a single, initial resting unoccluded blood flow measurement. In this study, blood flow at each set pressure was more appropriately expressed relative to a paired, resting unoccluded blood flow measurement.

In both males and females, relative blood flow was statistically similar between set pressures of 300 and 400 mmHg in the arm (Figure 2), and between 200 and 300 mmHg in the leg (Figure 3). Because the overall trend is a decrease in relative blood flow with increasing set pressures, this appears to be an isolated non-significant pairwise comparison rather than a true physiological plateau in blood flow. This is contrary to a previously reported trend in absolute blood flow (mL/min) that did not decrease with increasing pressures (14). There were no significant differences in blood flow (mL/min) between each measured pressure of 200, 250, 300, 350, and 400 mmHg and only blood flow at set pressures of 350 and 400 were significantly different than unoccluded blood flow (14).

The non-significant differences in blood flow at set pressures of 300 and 400 mmHg in the arm (Figure 2), and between 200 and 300 mmHg in the leg (Figure 3) may be due to the elastic properties of the B-Strong bands. At lower set pressures the bands are more compliant and blood flow restriction may be more similar between set pressures. At higher pressures, the band becomes less compliant, thereby increasing blood flow restriction (5). The similarities in relative blood flow at set pressures of 200 and 300 mmHg in the leg may be due to the large circumference of the leg and the need for higher set pressures to restrict blood flow when using soft, elastic, “multi-chambered” BFR bands that are designed to avoid complete arterial occlusion (5).

### 4.3 Implications of using soft elastic B-strong bands

The inability to achieve an AOP and the wide range of blood flow responses across the range of set pressures (Figure 1) present challenges to prescribing exercise with BFR using B-Strong bands. One of the reasons for using an individualized AOP-based set pressure during BFR training is to reduce the likelihood of complete arterial occlusion. Two implications of the data presented from this study are, first, users of B-Strong bands will not be able to follow AOP-based recommendations (1, 8–10) to select set pressures during BFR training. Second, even though the inability to completely occlude arterial blood flow is touted as a safety feature of the B-Strong cuffs, practitioners should be aware of the wide range of blood flow responses (Figure 1) to BFR between individuals. This data suggests that inflating the cuff to the same set pressure may not produce the same physiological stimuli across individuals. Thus, for some individuals, BFR training with B-Strong bands may not produce the anticipated outcome, while others may be at increased risk of adverse outcomes.

The physiological stimulus of BFR training is related to the reduction of arterial blood flow and venous return. This study only measured arterial blood flow. Without measures of venous blood flow, muscle oxygenation, or metabolic markers, the physiological stimulus of BFR cannot be fully ascertained. Practitioners can employ various strategies to overcome the challenge of selecting an appropriate set pressure during training. For example, the manufacturer recommends individualizing set pressure by using the recommended initial starting set pressure based on the selected length of the cuff, then adjusting the set pressure during subsequent exercise sessions based on repetitions completed and perceptions of fatigue or using the manufacturer-provided app (https://bstrong.training). Practitioners can implement a familiarization period to identify set pressures that provide an adequate metabolic stimulus as evidenced by onset of fatigue and venous distention (2, 5). Practitioners can also monitor perceptions of fatigue and repetitions to failure throughout the training session. Adjustments to set pressure can be made in subsequent training sessions (2, 5). Practitioners and BFR enthusiasts alike should also be able to recognize side effects and adverse events that may occur during or following training with BFR and adjust training parameters accordingly (2).

Although the reduction in blood flow is the underlying premise for BFR training adaptations, there are other related and interacting variables, such as the compression of the limb, tension due to muscle contraction, ischemia, hypoxia, venous blood flow restriction, metabolic stress, neural factors, motor unit recruitment, and muscle fatigue that contribute, perhaps synergistically, to the adaptations to resistance training with BFR (29, 30). Despite the wide range of blood flow responses to set pressures observed in this study and the presumed variability in training outcomes, the use of B-Strong bands during training has been effective in achieving significant muscle adaptations (5, 6).

The blood flow responses of the 60 participants in this study may not be generalizable to other males and females, or patients in clinical settings (e.g., injured, post-operative, de-conditioned, in pain). The blood flow responses of those who are accustomed to habitual exercise, resistance training, or using BFR during resistance training may be different. Blood flow responses to BFR may be related to training status, muscle mass, limb composition, vascular adaptations, neural responses and control of heart rate and blood pressure. We measured blood flow while the participants were in a resting, semi-reclined position. Blood flow responses to BFR may be different in postures of exercises performed when using BFR (e.g., standing). The blood flow response to BFR at rest when there are no muscle contractions do not represent the blood flow responses that occur during exercise. During muscle contractions, the elastic bands can accommodate the dynamic expansion of skeletal muscle which may attenuate blood flow responses (5). In this study we only evaluated the blood flow response to increasing set pressures using B-Strong bands. We did not compare the blood flow responses to BFR with B-Strong bands to the responses to BFR using other soft, elastic bands (e.g., KAATSU bands; https://kaatsu.com).

Although the interface pressure between a nylon single bladder cuff and the limb has previously been reported (31), investigating the interface pressure between a multi-chambered band and the limb would enhance our understanding of the blood flow responses to using multi-chambered BFR bands. Since it is feasible that the blood flow responses to BFR may be different in those individuals who regularly use BFR training systems, future studies can evaluate and compare the blood flow responses to different BFR set pressures between a group of participants who regularly use BFR training and a group of participants who have never used BFR. Similarly, investigating the pre-training blood flow responses to BFR set pressures to the blood flow responses following several weeks of BFR training would be informative.

## Data Availability

The raw data supporting the conclusions of this article will be made available by the authors, without undue reservation.
